# A Prospective Cohort Study on the Periparturient Muscle Tissue Mobilisation in High Producing Dairy Cows

**DOI:** 10.3390/ani12141772

**Published:** 2022-07-11

**Authors:** Cara Hatfield, William Tulley, Rachel Hall, Bethany Eloise Griffiths, Andreas Foskolos, Robert Frank Smith, Georgios Oikonomou

**Affiliations:** 1 Department of Livestock and One Health, Institute of Infection, Veterinary & Ecological Sciences, University of Liverpool, Neston CH647TE, UK; c.l.hatfield@liverpool.ac.uk (C.H.); w.j.tulley@liverpool.ac.uk (W.T.); hlrhall@liverpool.ac.uk (R.H.); hlbgriff@liverpool.ac.uk (B.E.G.); robsmith@liverpool.ac.uk (R.F.S.); 2 Department of Animal Science, University of Thessaly, 41500 Larisa, Greece; afoskolos@uth.gr

**Keywords:** periparturient, NEB, NEFAs, muscle thickness, BCS, ultrasound

## Abstract

**Simple Summary:**

The objectives of this study were to (i) investigate the changes in muscle tissue reserves in high producing dairy cows before and after calving, (ii) identify factors associated with these changes, and (iii) describe their possible associations with cattle reproductive performance. Data were collected from 455 cows on three different UK farms. Holstein cows mobilise both fat and muscle tissue reserves before and after calving. Significant differences in the amount of muscle mobilised were identified between farms; this could have been associated with pre calving diets. Higher genetic merit for milk yield was associated with lower muscle tissue reserves. An increased time to first service was described for those animals that mobilised more muscle tissue.

**Abstract:**

Excessive periparturient fat mobilisation and its association with dairy cattle health and fertility is well documented; however, the role of muscle mobilisation has not been studied extensively. The objectives of this study were to (i) investigate the changes in the thickness of the longissimus dorsi muscle in high producing dairy cows during the periparturient period, (ii) identify factors associated with these changes, and (iii) describe their possible associations with cattle reproductive performance. Data were collected from a total of 500 lactations from 455 cows on three different UK farms. Muscle thickness (MT) (*Longissimus dorsi*) and back fat thickness (BFT) measurements were collected at three different time-points during the periparturient period using ultrasonography. Body condition score (BCS) was also assessed at the same time-points and blood samples were collected for the measurement of non-esterified fatty acids. Farm fertility records were used and genomically estimated breeding values were also available. Associations between variables were analysed with the use of multivariable linear and logistic regression models; Cox proportional hazard analysis was used for fertility outcomes. Muscle thickness decreased pre- to post-calving on all three farms, though they were notable between farm differences. Those animals with a lower BCS pre-calving had a higher MT loss; significant fat mobilisation occurred between the calving and early lactation period. Muscle thickness changes and fat mobilisation were not associated in this study. An increased time to first service was described for those animals that mobilised more muscle tissue. Our study advances the understanding of periparturient muscle tissue mobilisation in dairy cattle and highlights its potential associations with cattle fertility.

## 1. Introduction

Dairy cattle face a drastic increase in energy demand at the onset of lactation [1]. This coincides with a drop in feed intake in the days surrounding parturition [2]. This imbalance of energy intake and demand leads to a period of negative energy balance (NEB) [1]. To meet these nutrient requirements, animals in early lactation rely on the metabolism of their body reserves (homeorhesis) [3]. Adipose tissue is mobilised in the form of non-esterified fatty acids (NEFAs) to meet energy demands for milk production [1]. Muscle tissue can also be mobilised to provide glucogenic amino acids for gluconeogenesis [4].

Excessive periparturient fat mobilisation and its association with dairy cattle health and fertility is well documented [5,6,7]. However, the role of muscle mobilisation has not been studied extensively with previous studies being limited to small numbers of Holstein cows in single herds or experimental settings [8,9,10]. These authors identified a consistent pattern of muscle catabolism in the late dry period and early lactation, with initiation commencing prior to the mobilisation of body fat. Whilst NEB is one potential reason for skeletal muscle degradation, there are several other potential causes. Nutritional causes can include specific protein or amino acid deficiencies [11]; evidence of negative protein balance during the periparturient period does exist [12]. Physiological causes include high plasma glucocorticoid levels [13], paresia, and insulin resistance [14]. Insulin resistance in humans can be due to pregnancy [15], obesity or physical inactivity [16]. Importantly, the possible associations of excessive muscle catabolism with dairy cattle health and fertility have not been studied yet. 

The main objectives of this prospective cohort study were to (i) investigate the changes in the thickness of the longissimus dorsi muscle in high producing dairy cows during the periparturient period, (ii) identify factors associated with these changes, and (iii) describe their possible associations with cattle reproductive performance.

## 2. Materials and Methods

The study was approved by the University of Liverpool Veterinary Research Ethics Committee (Reference Number: VREC269). Blood sampling was performed for the purposes of a different project and under a Home Office Project License.

Three farms were selected due to their proximity to the University of Liverpool Leahurst campus. A first pilot study was conducted from December 2014 until October 2015 on one of the three farms (Farm 1). The main study commenced on November 2016 and concluded August 2017. The three farms were all milking Holstein cows and varied in management and nutrition strategies. 

Farm 1 milked cows in a herringbone parlour three times daily and recorded approximately 12,300 kg of milk per cow per year. Lactating cows were housed in sawdust bedded cubicle sheds. Dry cows were housed in sheds with a deep straw lying area and a grooved concrete loafing area. Following parturition, fresh cows were also housed in loose straw yards (for 2 to 4 weeks). Farm 2 milked cows in a swing-over herringbone parlour twice daily and recorded approximately 9000 kg of milk per cow per year. Lactating cows were housed in sand bedded cubicle sheds. Dry cows were housed in sheds with a deep straw lying area and grooved concrete loafing area. Farm 3 milked cows in a rotary milking parlour three times daily and recorded approximately 12,000 kg of milk per cow per year. All cows throughout the lactation cycle were housed in deep sand bedded cubicles and grooved concrete loafing areas. Cows on all farms were dried off approximately 60 days before expected calving.

All farms fed dry and milking cows with a total mixed ration (TMR) along a feed passage; additional in-parlour concentrate feeding for yield occurred on Farm 2. Dry cows’ TMR on Farm 1 consisted of grass silage, maize silage, barley straw, haylage, rapeseed, and a mineral mix. Dry cows’ TMR on Farm 2 consisted of grass and maize silage, wheat straw, Trafford syrup, distillers’ grains wheat, and a mineral mix. Dry cows’ TMR on Farm 3 consisted of wholecrop wheat, wheat straw, rapeseed, urea, and a mineral mix. The milking cows’ diets on Farm 1 consisted of grass silage, haylage, maize silage, alkalage, wheat straw, barley, a concentrates blend, and a mineral mix. The milking cows’ diets on Farm 2 consisted of grass silage, maize silage, wheat straw, bread, Trafford syrup, caustic wheat, urea, distillers’ grains wheat, fat, and a minerals mix. The milking cows’ diets on Farm 3 consisted of maize silage, grass silage, wholecrop wheat, Trafford gold, molasses, bread, fat, rapeseed, soya meal, soya hulls, distillers’ grain wheat, barley, salt, urea, and a minerals mix. 

Data were collected from each animal on three occasions: Three to four weeks before the expected date of parturition, 0–10 days postpartum, and approximately 60–80 days postpartum, referred to as pre-calving, fresh and early lactation time-points, respectively. 

At each time-point, a trained researcher recorded body condition score (BCS), muscle thickness (MT), and back fat thickness (BFT). Body condition score was scored according to the Penn State assessment method. Scores range from 1 to 5 in 0.25 increments (1 = very thin, 5 = obese) [17]. Muscle and fat thickness were measured using an EASI-SCAN ultrasound machine (sonographic B-mode, BCF™ Technology, UK) equipped with a linear probe 5–8 MHz. Longissimus dorsi depth was measured perpendicularly to the skin at the fourth lumbar process, as described by van der Drift et al. (2012) [10]. Back fat thickness was also measured as described by van der Drift et al. (2012) [10].

Mobility score was assessed using the Agriculture and Horticulture Development Board (AHDB) Dairy Mobility Scoring system [18]. Cows were scored with 0 when they walked with even weight bearing and rhythm on all four feet, and with a flat back. Cows that stepped unevenly or had shortened strides were scored as 1; affected limb or limbs were not immediately identifiable. Cows with a score 2 had an uneven weight bearing on a limb or limbs that was immediately identifiable and/or clearly shortened strides (usually with an arch to the centre of the back). Score 3 cows were unable to walk as fast as a brisk human pace and had signs of score 2. At the early lactation time-point, all four feet were lifted and were visually inspected for the presence of claw horn disruption lesions (sole ulcers (SU), sole haemorrhage (SH), and white line disease (WLD)). The assessor and date of each measurement were also recorded. Each farm was visited by the researchers once per week.

Blood samples were collected from the coccygeal vein using 10 mL plain blood tubes (BD Vacutainer^®^). Blood samples were allowed to clot and then maintained on ice until transported to the laboratory within a few hours and centrifuged at room temperature for 2400 rpm x 10 min (PrO-Vet, Centurion Scientific Ltd., Chichester, UK). The serum was then separated and transferred into 2 mL Eppendorf tubes and stored at −20 °C until analysis. Non-esterified fatty acids (NEFA) concentrations were measured using a commercially available kit (NEFA: FA 115 kit, Randox Laboratories Ltd, London, UK). A modified method was utilised [19] to allow a 96-well plate and reader to be used. The optical density values were read using a microplate reader at a wavelength of 550 nm (MultiSkan EX Original, Thermo Fisher Scientific, Waltham, MA, U.S.) with the colour change as linearly proportional to the NEFA concentration.

In total, six researchers were involved in the data collection. Blood samples and BFT measurements were not available for the initial, pilot study. Blood sampling was always conducted at the same time of day for non-milking animals, and immediately after milking in milking cows. Therefore, sampling times relative to feed access were consistent within groups.

Fertility data (calving dates, insemination dates, and pregnancy diagnosis out-comes) were recorded by each farm on their separate recording systems. All farms had a voluntary waiting period of 45 days after which they would perform oestrus detection to inseminate cows. Farms 1 and 3 performed oestrus detection aided by activity monitoring devices (pedometers were used on Farm 1 and neck collars on Farm 3). Farm 2 used tail chalking. Cows on Farm 3 were enrolled on a timed artificial insemination protocol if not inseminated by 70 days post-calving. Genomically estimated predicted transmitting abilities (gPTAs) for milk production and for the fertility index were also available as the enrolled cows had already been genotyped for a different project [20].

## 3. Statistical Analysis

Data were analysed using JMP Pro 15 (SAS Institute Inc., Cary, NC). Descriptive and univariable analyses were undertaken between variables, before multivariable regression models were constructed. Parity was fitted in all models as a categorical variable with three levels (1 for cows in their first parity, 2 for cows in their second parity, and 3 for cows in their third or greater than third parity). Body condition score was also turned into a categorical variable with three levels (level 1 for BCS < 2.5, level 2 for BCS from 2.5 to 3, and level 3 for BCS > 3). NEFAs measurements immediately post-calving (fresh) were turned into a binary variable (0 for NEFA < 0.82 and 1 for NEFA ≥ 0.82 mmol/L; this was the median for NEFAs measurements in this population). Changes in MT between different assessment time-points were calculated and grouped in three equally sized groups; gPTAs were also grouped in three equally sized groups to facilitate data analysis and interpretation of results (1 for the group with the lowest gPTAs for a certain trait, to 3 for the group with the highest gPTAs).

Two separate mixed effects multivariable linear regression models were used to describe MT and BFT measurements. Explanatory variables were offered to the models due to the fact that they were associated with the outcome variable in univariable analyses (*p* ≤ 0.20) or they were important for this study. Cow identification was fitted in the model as a random effect to account for repeated measurements per animal and within animal clustering. The covariance structure used was compound symmetry. Associations between various explanatory variables were investigated to identify potential collinearity issues. All two-way interaction terms were also offered to these models. A manual stepwise approach was taken to remove variables and their interactions from the model (the variable with the highest *p*-value was removed at each step). Only variables with *p* < 0.10 (F test) were maintained in the final models. If an interaction term was found to be significant, then the main effects were also maintained in the final model whether they were significant or not. Rows with missing data were included in the analysis using the informative missing function in JMP. Residuals by model predicted values, studentized residuals, and residuals normal quantile plots were visualised to evaluate the model’s goodness of fit and that assumptions of normality and homoscedasticity were met. Partial-regression residual leverage plots for all fixed effects included in the models were also visualised. Results per categorical explanatory variables are presented as least-squared means ± standard error of the mean. Pairwise comparisons of least-squared means were performed using the Tukey-Kramer honestly significant difference (HSD) test. A linear regression model with the change in MT from the pre-calving to the fresh time-point (MT pre-calving to fresh) as an outcome was also built in a similar manner (but without fitting a random effect).

A multivariable logistic regression model with NEFAs concentration at fresh (binary) as an outcome was also built. Variables with a *p* *≤* 0.20 in the univariable analyses were offered to this multivariable logistic regression model. Variables were removed from the model manually and in a stepwise manner (with the variable with the highest *p*-value removed at each step), and only variables with *p* < 0.10 (likelihood ratio test) were maintained in the final model. The lack of fit test was used to evaluate the model’s goodness of fit and the likelihood ratio test was used to determine the overall significance of the model. The predictive ability of the final logistic regression model was assessed with receiver operating characteristic analysis and the calculated area under the curve. Results from this logistic regression model are presented as odds ratios. *p*-Values and 95% confidence intervals (CI) for calculated odds ratios (OR) are Wald based estimates. 

Cox proportional hazard analysis with right censoring was used to explore the effect of MT changes and other relevant explanatory variables on the intervals from calving to the first insemination and from calving to pregnancy.

## 4. Results

A total of 455 cows were enrolled with 45 cows from Farm 1 followed over two consecutive lactations. Therefore, data were collected for 500 lactations (312 from Farm 1, 75 from Farm 2, and 113 from Farm 3). One hundred and forty-four animals were studied in their first lactation, 134 in their second, and 222 in their third or greater lactation. Additional descriptive statistics information for the same study population has been presented by Griffiths et al. (2020) [20].

Explanatory variables remaining in model 1 (MT as an outcome) were: Time-point of measurement, parity group and its interaction with time-point, farm and its interaction with time-point, and milk gPTA category. Model’s R^2^ was 0.76. The random effect of cow accounted for 52.39% of the total variance. Adjusted means (least-squared means), standard errors, Tukey HSD, and *p*-values for different levels of explanatory variables are presented in Table 1. Assessor was also found to have a significant effect on MT (*p* < 0.0001) and was maintained in the model. Muscle thickness was decreasing in the period between the pre-calving and fresh measurement. This decrease was greater in cows maintained on Farm 3. Cows in the lowest milk PTA category had higher MT measurements compared to the other two categories, but there was no significant milk PTA category by time interaction. Muscle thickness was also lower in second parity cows (compared to first parity or greater than second parity cows).

To further investigate what was associated with the observed decrease in MT occurring between the pre-calving and fresh measurements we built a model with MT pre-calving to fresh as an outcome. Explanatory variables that remained in this model were: MT pre-calving, assessor (the combination of assessor at pre-calving and fresh), pre-calving BCS (grouped), days between pre-calving and fresh measurement and farm. Adjusted means (least-squared means), standard errors, Tukey HSD, and *p*-values for different levels of these explanatory variables are presented in Table 2. Adjusted means ± SE for MT pre-calving to fresh for each farm was −4.38 ± 2.27, −4.47± 2.41, and −6.81 ± 2.36 for Farms 1, 2, and 3, respectively (*p* < 0.001). A lower pre-calving BCS was associated with increased MT loss. For every 1 mm of muscle thickness pre-calving, an estimated loss of 0.45 mm ± 0.039 occurred in the pre-calving to fresh period. An increased length of time between the pre-calving to fresh measurement resulted in greater MT loss.

**Figure 1:**
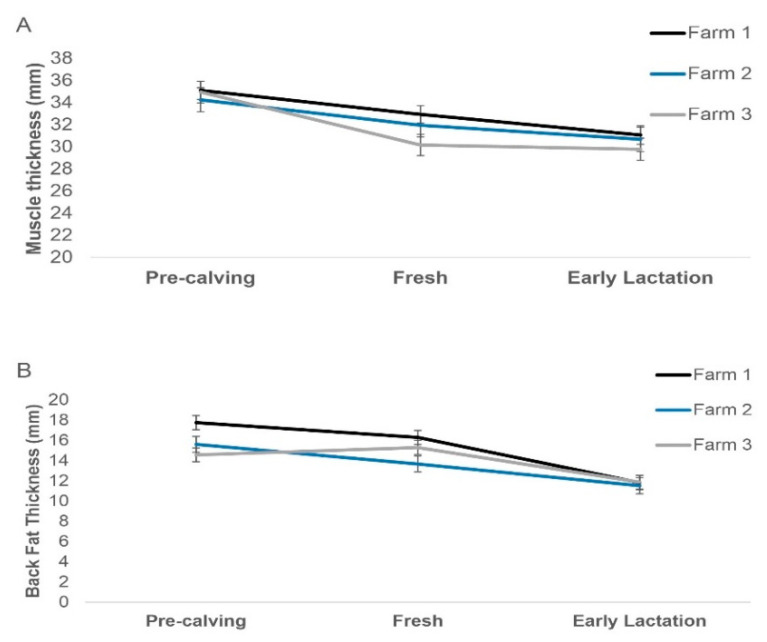
Adjusted means (±standard error) for muscle thickness (**A**) or back fat thickness (**B**) at different time-points and for the three different farms enrolled in this study.

Explanatory variables remaining in the model with BFT as an outcome were: Time-point of measurement, parity group and its interaction with time-point, farm and its interaction with time-point, and milk PTA category. Adjusted means (least-squared means), standard errors, Tukey HSD, and *p*-values for different levels of these explanatory variables are presented in Table 3. Significant fat mobilisation occurred in the period between the fresh and the early lactation measurements. Cows in the lowest milk PTA category had higher BFT measurements compared to the other two categories, but there was no significant milk PTA category by time interaction. Results regarding the interesting farm time-point interaction for both MT and BFT are also presented in Figure 1.

The logistic regression model with NEFAs at fresh (binary) as an outcome included the following explanatory variables: Farm, BCS pre-calving (grouped), and parity group (Table 4). MT loss pre-calving to fresh was not associated with increased NEFAs indicating that MT measurements represented muscle mass changes and not mobilisation of intra-muscle fat. This was re-enforced by the non-significant associations between MT and BFT changes (data not shown). Farm 3 had greater odds of having higher NEFAs at fresh compared to Farms 1 and 2 (OR: 39.76, CI = 15.45–102.14, *p* < 0.0001 and OR: 9.68, CI = 4.04–23.21, *p* < 0.001, respectively). Later, parity cows had greater odds of high NEFA concentrations than first and second parity cows (OR: 2.48, CI = 1.00–6.19, *p* = 0.05 and OR: 2.50, CI = 1.07–5.83, *p* = 0.034, respectively). Cows with a BCS pre-calving of >3 were at higher odds of increased NEFAs at fresh compared to those with a BCS of 2.5–3 (OR: 5.69, CI = 2.42–13.37, *p* < 0.001).

Explanatory variables that remained in the Cox proportional hazard model with time to first service as an outcome were: Farm (*p* = 0.004), fertility index category (*p* = 0.02), and MT pre-calving to early lactation category (*p* = 0.025). The hazard ratio for cows in the second MT pre-calving to early lactation category versus cows in the first category (cows with the greater loss of MT) was 1.45 (1.11–1.92, *p* = 0.007). The hazard ratio for cows in the third MT pre-calving to early lactation category (cows that had minimal loss of MT or gained MT during the studied period) versus cows in the first category was 1.28 (0.96–1.69, *p* = 0.09). Specifically, cows in the greatest MT loss group took significantly more time to reach their first insemination event. The hazard ratio for cows in the third fertility index category versus cows in the first category was 1.42 (1.07–1.88, *p* = 0.015).

Multivariable Cox proportional hazard analysis indicated that calving to conception interval was associated with parity group, farm, presence of SU in early lactation, and fertility index category. Cows in the third category for fertility index (best genetics for fertility) had lower calving to conception interval compared to cows in the first category (hazard ratio: 1.73, CI = 1.11–2.68, *p* = 0.015). Cows that did not have a presence of a SU also had lower calving to conception interval compared to cows with a SU in early lactation (hazard ratio: 1.76, CI = 1.00–3.29, *p* = 0.048). Primiparous animals reached pregnancy faster than multiparous ones (parity group 1 versus 2: Hazard ratio of 1.98, CI = 1.31–3.01, *p* = 0.0013; parity group 1 versus 3: Hazard ratio of 2.64, CI = 1.80–3.86, *p* = <0.0001). Cows in Farm 3 reached pregnancy faster compared to cows in Farm 1 (hazard ratio: 1.87, CI = 1.17–3.02, *p* = 0.0084).

## 5. Discussion

In this study, we demonstrate that MT decreased before and after calving in agreement with previous studies [10] and that significant between farm differences in the way muscle tissue was catabolised, which would not have been identified by body condition scoring (although this could partially be due to different assessors scoring the cows), did exist. Although body condition scoring is an acceptable method to help quantify adipose tissue [21] and does incorporate skeletal muscle [22], the use of US thickness measurements of the longisimus dorsi muscle, as used in our study, provides a more reliable measurement of muscle mobilisation [22]. The MT loss was not clearly associated with fat mobilisation within this study. Moreover, we demonstrate for the first time that excessive muscle catabolism in the periparturient period may be associated with detrimental effects on cattle fertility.

Mobilisation of fat stores is well recognised to occur in response to negative energy balance and happens predominantly postpartum [23]; our results are consistent with that. Asynchrony of muscle tissue mobilisation suggests an alternative mechanism or nutrient requirement, and has previously been documented in the postpartum period [24]. Higher body condition and fat levels prior to calving are associated with increased insulin resistance [25], lower dry matter intake [26] and increased mobilisation of body fat postpartum [27]. These processes might be expected to result in increased muscle mobilisation due to increased negative energy balance, or the requirement for amino acids to act as anaplerotic intermediaries. Mann et al. (2016) [4] did in fact identify increased muscle autophagy in cows that were overfed relative to energy requirements in the transition period. In our study, increased MT was associated with increased mobilisation of muscle tissue, but increased BFT was not. Van der Drift et al. (2012) [10] showed cows mobilising increased amounts of skeletal muscle–identified by higher 3-methylhistidine concentration–had lower beta-hydroxybutyrate concentrations, suggestive of a protective effect on fat mobilisation, or more complete oxidation of mobilised NEFA; however an association between NEFAs concentration immediately after calving and muscle mobilisation did not exist in our study.

The initiating factors for mobilisation of skeletal muscle and the fate of the mobilised amino acids are unclear and investigating this was beyond the scope of this study. Demand for amino acids is increased both prior to calving for mammary development, fetal growth, and colostrogenesis and post-calving for milk protein synthesis [12], whilst requirements for the immune system must also be satisfied. Increased nutrient demand is typically compounded by the decline in dry matter intakes in the periparturient period [2] accompanied by transient insulin resistance, in order that the available nutrients are prioritised for milk production. In this situation, amino acids can also be used as an energy source via gluconeogenesis, analplerosis, and ketogenesis. However, in agreement with the findings of previous studies [9,10,24] the asynchrony between fat and protein mobilisation indicates that negative energy balance is not the sole or primary initiator of mobilisation of skeletal muscle in the peripartum period. 

Both animal and environmental factors influence MT, with differences between farms and parities. Second lactation animals have lower MT than heifers or mature cows [28], and it is likely that this occurs due to failure to re-accrete muscle tissue between first and second calving due to requirements for lactation and growth. Farm effects may be associated with differences in nutritional management, both during lactation and the dry period, with prior evidence that dietary manipulation during the dry period can influence longissimus dorsi thickness [28]. For the two farms, for which detailed dry period diet information was available in this study (data not shown), predicted metabolisable protein (MP) yield was lower in the farm that had greater MT loss over the study period, suggesting that increased MP may be protective against decrease in MT and potentially supplies an alternative source of amino acids. Further research is required to determine if this is a valid conclusion.

Higher genetic merit for milk yield has been found to be associated with greater lipolysis due to an increase in activity of hormone-sensitive lipase and a greater response to β-adrenergic stimulation [29,30]. Multiparous animals in our study were found to have a greater risk of higher NEFAs at fresh in comparison to primiparous, which is not unexpected due to their greater milk production. However, milk PTA was not associated with high NEFAs at fresh in our study. Genetic merit for milk yield was still associated with differences in body composition, with animals in the lowest category for PTA milk having both increased BFT and MT, suggesting that these animals may preferentially partition nutrients to body reserves, rather than milk production. 

Fertility index was favourably associated with days to first service and calving to conception interval supporting the importance of genetic selection for the improvement of Holstein cows’ reproductive performance. Days to first service were also influenced by farm, suggesting environmental and management effects, but were also associated with the degree of change in MT with cows with the least loss in muscle tissue having the lowest days to first service. An unfavourable association was found between cows that lost the greatest MT over the entire study period (pre-calving to early lactation) and days to first service. This suggests that reproductive performance could be poorer when muscle mobilisation occurs over an extended period. Our study does not provide an explanation of a mechanism for the relationship between muscle catabolism and fertility, but this warrants further investigation as a potential mechanism to improve reproductive performance in higher producing dairy cows.

Variation in assessor was found to be significant and thus is a potentially confounding factor, suggesting imperfect agreement between different operators. All assessors were trained by the same experienced researcher and according to a previously described method [10], but an assessment of agreement between different assessors was not undertaken. In future studies, capture and analysis of ultrasound images should ideally be limited to a single operator. Alternatively, a biochemical marker of muscle mobilisation, such as 3-methylhistadine [31] or creatinine [9] as an indicator of total skeletal muscle mass, could be used; however these are only markers of muscle mobilisation and not necessarily more accurate than repeated ultrasound measurements. Further limitations of our work include the inability to correct for the confounding effect of assessor variation when performing fertility analysis with MT loss as an explanatory variable. The overall effect of MT changes on fertility could be more or less influential than currently presented. Although not a focus in this study, diet does play a role in body composition and nutritional management may be influential on the study findings and could explain part of the significant farm effect described here. As this study was designed as observational, an alternative approach would be to conduct a randomised control trial to allow ration alterations. 

## 6. Conclusions

In agreement with previous studies, we have identified significant mobilisation of body fat and muscle tissue across the transition period in dairy cows. Asynchrony in mobilisation of fat and muscle suggests that negative energy balance is not the sole underlying cause of muscle catabolism, and that a variety of different uses are possible for the mobilised amino acids. Furthermore, we have identified an association between the degree of muscle catabolism and subsequent reproductive performance. Variation in genetics, diet, and environment at individual and herd level provide potential mechanisms to manipulate the amount of muscle catabolised in the peripartum period and thus further study is warranted in these areas.

## Figures and Tables

**Table 1:** Results from a multivariable mixed effects linear regression model for outcome muscle thickness (mm). HSD: Honestly significant difference; levels within a variable with different letters are statistically significantly different (*p* < 0.05).

Variable	Level	Adjusted Mean	Std Error	Tukey HSD	*p*-Value
Farm	1	33.05	0.68	A	0.042
	2	32.31	0.91	AB	
	3	31.64	0.81	B	
Time-point	Pre-calving	34.78	0.83	A	<0.001
	Fresh	31.70	0.81	B	
	Early Lactation	30.52	0.86	B	
Parity group ^1^	1	32.33	0.70	A	0.002
	2	30.71	0.72	B	
	3	32.09	0.67	A	
Milk PTA category ^2^	1	34.01	0.67	A	0.004
	2	31.85	0.67	B	
	3	31.82	0.66	B	
Parity group ^1^ by Time-point	1, Pre-calving	34.78	0.83	AB	<0.001
	1, Fresh	31.70	0.81	CD	
	1, Early Lactation	30.52	0.86	CDE	
	2, Pre-calving	33.28	0.86	BC	
	2, Fresh	30.09	0.85	DE	
	2, Early Lactation	28.77	0.87	E	
	3, Pre-calving	35.75	0.78	A	
	3, Fresh	31.72	0.76	CD	
	3, Early Lactation	28.80	0.80	E	
Farm by Time-point	1, Pre-calving	35.12	0.82	A	0.003
	1, Fresh	32.95	0.79	ABC	
	1, Early Lactation	31.08	0.84	BCD	
	2, Pre-calving	34.26	1.09	AB	
	2, Fresh	31.97	1.08	ABCD	
	2, Early Lactation	30.69	1.10	CD	
	3, Pre-calving	34.96	0.97	A	
	3, Fresh	30.18	0.96	D	
	3, Early Lactation	29.79	1.00	D	

^1^ Parity group: 1 for cows in their first parity, 2 for cows in their second parity, 3 for cows in their third or greater parity. ^2^ Milk PTA category: 1 for the group with the lowest gPTAs for milk production to 3 for the group with the highest gPTAs.

**Table 2:** Results from a multivariable mixed effects linear regression model for outcome muscle thickness (mm) pre-calving to fresh (negative values describe loss of MT). Estimates for continuous describe the change in the outcome variable per one unit increase in the explanatory variable. HSD: Honestly significant difference; levels within a variable with different letters are statistically significantly different (*p* < 0.05).

Variable	Level	Adjusted Mean	Std Error	Tukey HSD	*p*-Value
Farm	1	−4.38	2.27	A	<0.001
	2	−4.47	2.41	A	
	3	−6.80	2.36	B	
BCS Pre-Calving Group ^1^	1	−13.98	4.46	AB	<0.001
	2	−6.26	0.58	B	
	3	−3.62	0.54	A	
Muscle Thickness Pre-Calving	Continuous Variable	Estimate −0.45	0.039		<0.001
Days between Pre-Calving and Fresh Muscle Measurement	Continuous Variable	Estimate −0.12	0.028		<0.001

^1^ BCS pre-calving group: 1 for BCS < 2.5, 2 for BCS from 2.5 to 3, 3 for BCS > 3.

**Table 3:** Results from a multivariable mixed effects linear regression model for outcome back fat thickness (mm). HSD: Honestly significant difference; levels within a variable with different letters are statistically significantly different (*p* < 0.05).

Variable	Level	Adjusted Mean	Std Error	Tukey HSD	*p*-Value
Farm	1	15.29	0.60	A	<0.001
	2	13.60	0.66	B	
	3	13.91	0.59	B	
Time-point	Pre-calving	15.98	0.62	A	<0.001
	Fresh	15.08	0.62	A	
	Early Lactation	11.74	0.63	B	
Milk PTA category ^1^	1	15.83	0.52	A	0.003
	2	13.87	0.49	B	
	3	14.08	0.43	B	
Farm by Time-point	1, Pre-calving	17.76	0.70	A	<0.001
	1, Fresh	16.29	0.69	AB	
	1, Early Lactation	11.82	0.71	D	
	2, Pre-calving	15.61	0.78	BC	
	2, Fresh	13.67	0.79	CD	
	2, Early Lactation	11.52	0.80	D	
	3, Pre-calving	14.57	0.68	BC	
	3, Fresh	15.29	0.70	BC	
	3, Early Lactation	11.87	0.69	D	
Parity group ^2^ by Time-point	1, Pre-calving	15.98	0.62	A	0.041
	1, Fresh	15.08	0.62	A	
	1, Early Lactation	11.74	0.63	B	
	2, Pre-calving	14.83	0.63	A	
	2, Fresh	14.51	0.64	A	
	2, Early Lactation	10.72	0.64	B	
	3, Pre-calving	16.01	0.58	A	
	3, Fresh	14.94	0.59	A	
	3, Early Lactation	10.17	0.60	B	

^1^ Milk PTA category: 1 for the group with the lowest gPTAs for milk production to 3 for the group with the highest gPTAs. ^2^ Parity group: 1 for cows in their first parity, 2 for cows in their second parity, 3 for cows in their third or greater parity.

**Table 4:** Results from a logistic regression model with NEFAs at fresh (binary, 0.82 mmol/l threshold) as an outcome. Presented odds ratios (OR) are for odds of having a NEFAs measurement higher than 0.82 mmol/l when comparing the level on the left of the pertinent column to the level on the right. *p*-Values and 95% confidence intervals (CI) for calculated odds ratios are Wald based estimates.

Outcome	Explanatory Variable	Levels		OR	95% CI	*p*-Value
NEFAs at Fresh	Farm	3 3	1 2	39.76 9.68	15.45–102.14 4.04–23.21	0.001
	BCS Pre-Calving group ^1^	3	2	5.69	2.42–13.37	0.00001
	Parity group ^2^	3 3	1 2	2.48 2.50	1.00–6.19 1.07–5.83	0.039

^1^ BCS pre-calving group: 1 for BCS < 2.5, 2 for BCS from 2.5 to 3, 3 for BCS > 3. ^2^ Parity group: 1 for cows in their first parity, 2 for cows in their second parity, 3 for cows in their third or greater parity.

## Data Availability

The datasets generated and analysed during the current study are available at reasonable request.

## References

[1] Herdt T.H. (2000). Ruminant adaptation to Influences on the Etiology of Ketosis and Fatty Liver. Vet. Clin. N. Am. Food Anim. Pract..

[2] Grummer R.R., Mashek D.G., Hayirli A. (2004). Dry Matter Intake and Energy Balance in the Transition Period. Vet. Clin. N. Am. Food Anim. Pract..

[3] Bauman D.E., Currie W.B. (1980). Partitioning of Nutrients during Pregnancy and Lactation: A Review of Mechanisms Involving Homeostasis and Homeorhesis. J. Dairy Sci..

[4] Mann S., Abuelo A., Nydam D.V., Leal Yepes F.A., Overton T.R., Wakshlag J.J. (2016). Insulin Signaling and Skeletal Muscle Atrophy and Autophagy in Transition Dairy Cows either Overfed Energy or Fed a Controlled Energy Diet Prepartum. J. Comp. Physiol. B Biochem. Syst. Environ. Physiol..

[5] Butler W.R., Smith R.D. (1989). Interrelationships between Energy Balance and Postpartum Reproductive Function in Dairy Cattle. J. Dairy Sci..

[6] Fenwick M.A., Llewellyn S., Fitzpatrick R., Kenny D.A., Murphy J.J., Patton J., Wathes D.C. (2008). Negative Energy Balance in Dairy Cows in Associated with Specific Changes in IGF-Binding Protein Expression in the Oviduct. Reproduction.

[7] O’Doherty A.M., O’Gorman A., al Naib A., Brennan L., Daly E., Duffy P., Fair T. (2014). Negative Energy Balance Affects Imprint Stability in Oocytes Recovered from Postpartum Dairy Cows. Genomics.

[8] Jaurena G., Moorby J.M., Fisher W.J., Cantet R. (2005). Association of Body Weight, Loin Longissimus Dorsi and Backfat with Body Condition Score in Dry and Lactating Holstein Dairy Cows. Anim. Sci..

[9] Megahed A.A., Hiew M.W.H., Ragland D., Constable P.D. (2019). Changes in Skeletal Muscle Thickness and Echogenicity and Plasma Creatinine Concentration as Indicators of Protein and Intramuscular Fat Mobilization in Periparturient Dairy Cows. J. Dairy Sci..

[10] van der Drift S.G.A., Houweling M., Schonewille J.T., Tielens A.G.M., Jorritsma R. (2012). Protein and Fat Mobilization and Associations with Serum β-Hydroxybutyrate Concentrations in Dairy Cows. J. Dairy Sci..

[11] Smith K., Rennie M.J. (1990). Protein Turnover and Amino Acid Metabolism in Human Skeletal Muscle. Bailliere's Clin. Endocrinol. Metab..

[12] Bell A.W., Burhans W.S., Overton T.R. (2000). Protein Nutrition in Late Pregnancy, Maternal Protein Reserves and Lactation Performance in Dairy Cows. Proc. Nutr. Soc..

[13] Schakman O., Kalista S., Barbé C., Loumaye A., Thissen J.P. (2013). Glucocorticoid-Induced Skeletal Muscle Atrophy. Int. J. Biochem. Cell Biol..

[14] Evans W.J. (2010). Skeletal Muscle Loss: Cachexia, Sarcopenia, and Inactivity. Am. J. Clin. Nutr..

[15] Vejrazkova D., Vcelak J., Vankova M., Lukasova P., Bradnova O., Halkova T., Kancheva R., Bendlova B. (2014). Steroids and Insulin Resistance in Pregnancy. J. Steroid Biochem. Mol. Biol..

[16] Mcglory C., Von Allmen M.T., Stokes T., Morton R.W., Hector A.J., Lago B.A., Raphenya A.R., Smith B.K., Mcarthur A.G., Steinberg G.R. (2017). Failed Recovery of Glycemic Control and Myofibrillar Protein Synthesis with Two Weeks of Physical Inactivity in Overweight, Pre-Diabetetic Older Adults. J. Gerontol. Ser. A.

[17] Ferguson J.D., Galligan D.T., Thomsen N. (1994). Principal Descriptors of Body Condition Score in Holstein Cows. J. Dairy Sci..

[18] Reader J.D., Green M.J., Kaler J., Mason S.A., Green L.E. (2011). Effect of Mobility Score on Milk Yield and Activity in Dairy Cattle. J. Dairy Sci..

[19] Miksa I.R., Buckley C.L., Poppenga R.H. (2004). Detection of Nonesterified (Free) Fatty Acids in Bovine Serum: Comparative Evaluation of Two Methods. J. Vet. Diagn. Investig..

[20] Griffiths B.E., Mahen P.J., Hall R., Kakatsidis N., Britten N., Long K., Robinson L., Tatham H., Jenkin R., Oikonomou G. (2020). A Prospective Cohort Study on the Development of Claw Horn Disruption Lesions in Dairy Cattle; Furthering Our Understanding of the Role of the Digital Cushion. Front. Vet. Sci..

[21] Schröder U.J., Staufenbiel R. (2006). Invited Review: Methods to Determine Body Fat Reserves in the Dairy Cow with Special Regard to Ultrasonographic Measurement of Backfat Thickness. J. Dairy Sci..

[22] Siachos N., Oikonomou G., Panousis N., Banos G., Arsenos G., Valergakis G.E. (2021). Association of Body Condition Score with Ultrasound Measurements of Backfat and Longissimus Dorsi Muscle Thickness in Periparturient Holstein Cows. Animals.

[23] Valergakis G.E., Oikonomou G., Arsenos G., Banos G. (2011). Phenotypic Association between Energy Balance Indicators and Reproductive Performance in Primiparous Holstein Cows. Vet. Rec..

[24] Tamminga S., Luteijn P.A., Meijer R.G.M. (1997). Changes in Composition and Energy Content of Liveweight Loss in Dairy Cows with Time after Parturition. Livest. Prod. Sci..

[25] De Koster J.D., Opsomer G. (2013). Insulin Resistance in Dairy Cows. Vet. Clin. N. Am. Food Anim. Pract..

[26] Treacher R.J., Reid I.M., Roberts C.J. (1986). Effect of Body Condition at Calving on the Health and Performance of Dairy Cows. Anim. Prod..

[27] Roche J.R., Berry D.P., Lee J.M., Macdonald K.A., Boston R.C. (2007). Describing the Body Condition Score Change Between Successive Calvings: A Novel Strategy Generalizable to Diverse Cohorts. J. Dairy Sci..

[28] Jaurena G., Moorby J.M. (2017). Lactation and Body Composition Responses to Fat and Protein Supplies during the Dry Period in Under-Conditioned Dairy Cows. J. Dairy Sci..

[29] McNamara J.P. (1989). Regulation of Bovine Adipose Tissue Metabolism During Lactation. 5. Relationships of Lipid Synthesis and Lipolysis with Energy Intake and Utilization. J. Dairy Sci..

[30] Smith T.H., McNamara J.P. (1990). Regulation of Bovine Adipose Tissue Metabolism During Lactation. 6. Cellularity and Hormone-Sensitive Lipase Activity as Affected by Genetic Merit and Energy Intake. J. Dairy Sci..

[31] Blum J.W., Reding T., Jans F., Wanner M., Zemp M., Bachmann K. (1985). Variations of 3-Methylhistidine in Blood of Dairy Cows. J. Dairy Sci..

